# In vitro responses to platelet-rich-plasma are associated with variable clinical outcomes in patients with knee osteoarthritis

**DOI:** 10.1038/s41598-021-90174-x

**Published:** 2021-06-01

**Authors:** Habib Zahir, Bijan Dehghani, Xiaoning Yuan, Yurii Chinenov, Christine Kim, Alissa Burge, Reyna Bandhari, Daniel Nemirov, Patrick Fava, Peter Moley, Hollis Potter, Joseph Nguyen, Brian Halpern, Laura Donlin, Lionel Ivashkiv, Scott Rodeo, Miguel Otero

**Affiliations:** 1 Hospital for Special Surgery, 535 E 70th Street, New York, NY 10021 USA; 2 New York Institute of Technology, Old Westbury, NY USA; 3 NewYork-Presbyterian Hospital, New York, NY USA; 4 The David Z. Rosensweig Genomics Research Center, New York, NY USA; 5 Columbia University, New York, NY USA; 6 HSS Center for Regenerative Medicine, New York, NY USA; 7 Derfner Foundation Precision Medicine Laboratory, New York, NY USA

**Keywords:** Genome-wide analysis of gene expression, Translational research, Osteoarthritis

## Abstract

Autologous blood-derived products such as platelet-rich plasma (PRP) are widely used to treat musculoskeletal conditions, including knee osteoarthritis (OA). However, the clinical outcomes after PRP administration are often variable, and there is limited information about the specific characteristics of PRP that impact bioactivity and clinical responses. In this study, we aimed to develop an integrative workflow to evaluate responses to PRP in vitro, and to assess if the in vitro responses to PRP are associated with the PRP composition and clinical outcomes in patients with knee OA. To do this, we used a coculture system of macrophages and fibroblasts paired with transcriptomic analyses to comprehensively characterize the modulation of inflammatory responses by PRP in vitro. Relying on patient-reported outcomes and achievement of minimal clinically important differences in OA patients receiving PRP injections, we identified responders and non-responders to the treatment. Comparisons of PRP from these patient groups allowed us to identify differences in the composition and in vitro activity of PRP. We believe that our integrative workflow may enable the development of targeted approaches that rely on PRP and other orthobiologics to treat musculoskeletal pathologies.

## Introduction

Osteoarthritis (OA) is a whole joint disorder and a major cause of pain and disability in adults. Inflammation is a known contributing factor to OA, and both local and peripheral inflammation are associated with disease activity, pain and knee stiffness. The complex pathogenesis of OA has resulted in significant challenges for the development of therapeutic strategies and currently there are few viable alternatives to total joint replacement. The available non-surgical interventions aim to delay disease progression, improve function, and treat pain by decreasing inflammation^1,2^.

The current standard methods available to clinicians to treat joint inflammation include oral non-steroidal anti-inflammatory medications and corticosteroid injection. However, these treatments are associated with variable outcomes, and as result clinicians have begun to use “orthobiologics” due to their potential to diminish inflammation and promote anabolism and repair, and because its “minimally manipulated” and autologous nature is not subjected to pre-market regulatory clearance from the Food and Drug Administration^3^.

Platelet-rich-plasma (PRP) is an autologous blood product generated by centrifugation of whole venous blood to isolate and concentrate platelets. It is believed that platelets, which contain and release numerous growth factors, promote healing, dampen inflammation, and reduce pain^3–5^. These blood-derived products have been used to enhance chondrogenesis^6,7^, treat bone^8,9^, tendon^10,11^ and ligament^12,13^ injuries, and have recently emerged as a potential therapeutic for knee OA^14^.

The available studies comparing intra-articular PRP injections to other means of non-surgical intervention for knee OA often report benefits for patients receiving PRP. The current view is that positive clinical results in OA patients are mainly related to the anti-inflammatory effects of PRP rather than to increased anabolic and reparative effects in articular cartilage^4,14^. However, these reports are inconclusive because of the high variability in the available PRP preparations, the limited available information about the relevant characteristics in these blood products that impact clinical responses in patients with knee OA, the lack of consensus and standardization of treatment protocols and collection of outcomes, and the poor understanding of disease candidates or specific cell/tissue targets for a given therapy^15^.

In the current study, we aimed to develop an integrative workflow to assess if the PRP composition and bioactivity in vitro is associated with clinical outcomes in patients with knee OA. Because of the well-characterized cellular crosstalk between synovial fibroblasts and macrophages^16^ and the contribution of these cells to OA disease^17,18^, we evaluated the anti-inflammatory actions of PRP in cocultures that reproduce the cellular crosstalk that occurs in inflamed synovial tissues. We characterized the responses to PRP in macrophages and fibroblasts using transcriptomics analyses. Relying on clinical outcomes, we identified knee OA patients with variable pain responses to intra-articular PRP injection. Using our bioassay and PRP from these patients we were able to detect differences in the PRP bioactivity that were associated with patient-reported outcomes.

## Patients and methods

### Subjects

The study protocol was approved by the Institutional Review Board (IRB) of the Hospital for Special Surgery (HSS). Written informed consent was obtained from all participants before entering the study, and the study and all methods were performed in accordance with the relevant guidelines and regulations. After IRB approval (HSS IRB#2016-0267) and patient consent, 51 knee OA patients were enrolled in our study upon presentation to clinic and eligibility for platelet-rich-plasma (PRP) injection, following the inclusion and exclusion criteria indicated in Supplementary Table S1, including Kellgren-Lawrence OA grade 1–3 based on plain radiographs. Knee OA patients received 1 intra-articular injection of leukocyte-reduced PRP. See Table 1 for patient demographics and PRP details. We collected patient-reported outcome measures (Knee injury and Osteoarthritis Outcome (KOOS-JR) and Numeric Pain Rating Scale (NPRS))^19,20^ at baseline, 6 weeks, and 6 months after PRP injection. For selection of PRP and downstream analyses, patients were categorized as responders (resp) or non-responders (n-resp) based on achievement of Minimal Clinically Important Difference (MCID) values (10-point increase for KOOS JR, and 20% decrease for NPRS)^21,22^ on the outcome measures reported at 6 months^23^. Additionally, we prepared PRP from healthy donors to obtain enough PRP volume to conduct in vitro experiments. Participants were enrolled in the study between 10/28/2017 and 11/26/2018.

**Table 1:** Patient demographics and platelet-rich-plasma characteristics.

Demographics	
Sex (male:female)	26:25
Age (years, mean ± s.d.)	57.92 ± 10.11
BMI (kg/m2, mean ± s.d.)	25.96 ± 4.07

Whole blood	
Platelets (× 10^3^/µl, mean ± s.d.)	226.30 ± 48.33
Leukocytes (× 10^3^/µl mean ± s.d.)	6.53 ± 1.96

Platelet-Rich-Plasma	
Kit	Arthrex, ACP
Time (minutes, mean ± s.d.)	4.3 ± 7.3
Vol prepared (ml, mean ± s.d.)	4.65 ± 0.22
Vol injected (ml, mean ± s.d.)	3.7 ± 0.7
Platelets (× 10^3^/µl, mean ± s.d.)	361.40 ± 67.41
Leukocytes (× 10^3^/µl, mean ± s.d.)	1.34 ± 0.81

### Platelet-rich-plasma (PRP) preparation

We used the Arthrex Autologous Conditioned Plasma kit series 1 (Arthrex, Naples, FL) for sample preparation. The characteristics of the PRP preparations, volume collected and injected, and time from collection to injection are summarized in Table 1 and reported following the MIBO guidelines^24^. Immediately after PRP preparation, patients received an intra-articular injection of PRP following standard procedures. The remaining PRP was immediately transported to the laboratory, treated with an additional 10% acid citrate dextrose solution A, aliquoted, and used for downstream analyses. One aliquot of PRP (200 µl) and one aliquot of whole blood collected at the time of the PRP preparation were used to determine the complete blood count (CBC) with differential in the Department of Pathology and Laboratory Medicine at HSS (see Table 1). Exogenous activation of PRP for in vitro experiments or ELISA assays was done using calcium chloride (CaCl_2_, final concentration 22.8 mM), as described^25^.

### Coculture system of macrophages and synovial fibroblasts

To evaluate the in vitro biologic activity of PRP, we used a previously described coculture system of primary human synovial fibroblasts and macrophages^16^. Fibroblasts were isolated as described^26^ from deidentified synovial tissues retrieved from patients undergoing total knee arthroplasty for rheumatoid arthritis (RA) or osteoarthritis (OA), after IRB approval and patient consent, and used for coculture experiments between passages 4 and 7, also as described^16^. Human CD14 + monocytes were freshly isolated from whole blood obtained from the New York Blood Bank and treated with 10 ng/ml of monocyte colony stimulating factor (M-CSF, PeproTech) for 24 h to promote a pro-macrophage lineage^27^. At 24 h after M-CSF treatment, macrophages and fibroblasts were cocultured in a 0.4 µm polyester trans-well system (Corning), as described^16^. The cocultured cells were left untreated (control, medium) or incubated with 10% v:v of activated PRP, 20 ng/ml of recombinant human TNFα (PeproTech), or PRP + TNFα for an additional 24 h. At 24 h, RNA was isolated for RNA-seq, NanoString, and RTqPCR analyses. For dose–response experiments, cells were left untreated or treated with 1%, 10%, and 25% of PRP (v:v) alone or combined with 20 ng/ml of TNFα for 24 h. Time-course experiments were performed using cells treated with 10% of PRP, alone or combined with TNFα, for 1 h, 6 h, and 24 h. For selected experiments, human recombinant IL-1β (R&D Systems, 10 ng/ml) was used instead of TNFα.

### RNA sequencing (RNA-seq)

Total RNA was extracted using the RNeasy mini kit (Qiagen) from cocultures of macrophages and fibroblasts, treated with the indicated conditions (control, PRP, TNFα, or PRP + TNFα) for 24 h. RNA-seq analyses were done in 4 independent experiments, using fibroblasts and macrophages obtained from 4 different tissue donors, and PRP samples prepared from 4 different subjects. A total of 100 ng of high-quality RNA (RIN > 8 and A260/280 > 1.8) were used to construct libraries. Sequencing was performed using an Illumina HiSeq 2500 at the Epigenomics Core Facility of Weill Cornell Medicine (50-bp single-end protocol) at a depth of 25 million mappable reads per sample. Read quality evaluation and adapter trimming was performed using fastp^28^. Read mapping to human genome (hg38) and counting against Gencode annotation was performed with STAR. Differential gene expression analysis was performed with edgeR using quasilikelyhood framework. Batch correction to account for the day of the experiment was performed by including a relevant batch term into a general-linear model. Genes with FDR-corrected p-values less than 0.05 and log2 fold change > 1 were considered differentially expressed. Downstream analyses were performed in R using a Shiny-driven visualization platform (RNAseq DRaMA) developed at the HSS David Z. Rosensweig Genomics Research Center.

### Quantitative set analysis of gene expression (QuSAGE) pathway analyses

To determine differentially regulated pathways we performed QuSAGE pathway analysis^29^ using MsigDB 6.2 C2 set (curated gene sets) as a reference^30^. QuSAGE approach relies on comparisons of differences between pathway-level probability density functions (PDF) for control and treatment samples. For a detailed description of the method, see^30^. Briefly, first, the PDF for the difference between control and treatment for each gene in the pathway is constructed by sampling from a standardized t-distribution with the degrees of freedom calculated from the data. Then, individual gene PDFs for all genes in a gene set are combined using numeric convolution to yield a pathway PDF. To account for the differences in pathway sizes, the pathways PDF is scaled by 1/N where N is the number of genes in a set. To account for correlations between genes, a pathway PDF is further scaled using variance inflation factor (VIF) that is estimated from the data. All gene sets with FDR-corrected *p* values < 0.05 were visualized using RNAseq DRaMA.

### Magnetic resonance imaging (MRI)

For patients with MRI obtained at HSS, MRI was performed using a cartilage-sensitive pulse sequence with an intermediate-weighted moderate echo time (TE) protocol that has already been assessed for accuracy based on an arthroscopic standard, with almost perfect agreement for reproducibility^31^. MRI readings of the affected knees were performed independently by two trained radiologists blinded to all other data using the Whole-Organ Magnetic Resonance Imaging Score (WORMS) method, as described^32^.

### nCounter NanoString gene expression analyses

For NanoString gene expression analyses, we used 100 ng of total RNA (RIN > 8, 260/280 > 1.8) isolated from 3 independent co-culture experiments, using macrophages and fibroblasts from 3 different blood and tissue donors, and PRP prepared from 3 different subjects. Analyses were performed using the nCounter PanCancer Human Pathways Panel (NanoString Technologies, Seattle, WA), following the manufacturer’s instructions. Data analysis was performed using the accompanying software (nSolver 4.0, NanoString Technologies, Seattle, WA).

### RTqPCR analyses

Reverse transcriptase quantitative polymerase chain reaction (RTqPCR) analyses of selected target genes were carried out using SYBR Green I-based real-time PCR, as described^33^, and specific primers against *CCL5*, *CXCL1*, *CXCL3*, *CYLD*, *IL1B*, *IL23A*, *MMP3, PPBP* and *GAPDH* (Supplementary Table S2). The data was calculated as the ratio of each gene to GAPDH.

### ELISA and multiplex ELISA

We performed magnetic bead-based sandwich immunoassays using the MILLIPLEX MAP multiplex Human Cytokine Panel 1 (cat #HCYTOMAG-60 k; EMD-Millipore Corporation, St. Charles, MO) following the manufacturer’s instructions. Duplicate wells of activated PRP samples were analyzed using a Luminex MagPix (Luminex). Cytokine concentrations were determined using the Luminex Xponent 4.2 and EMD-Millipore Milliplex Analyst v5.1. A human TGFb1 Quantikine ELISA Kit (R&D Systems) was used to evaluate TGFβ1 in PRP, using a Tecan Infinite M200 PRO plate reader, with the accompanying i-control 1.12 software (Tecan).

### Statistical analysis

Analyses of the patient-reported outcomes and multiplex ELISA assays were done at the Biostatistics Core of the Hospital for Special Surgery. Data were assessed for approximation to the Gaussian distribution using the D’Agostino-Pearson omnibus test of normality. Distributions were considered to be Gaussian if the P-value for the null hypothesis was greater than 0.05. Unpaired student *t*-test was used to compare two groups. Analyses of non-normally distributed data was performed using Mann–Whitney test. Statistical analyses of the qPCR results were performed using GraphPad Prism 8 (GraphPad Software, San Diego, CA). For data involving multiple comparisons, one-way analysis of variance (ANOVA) was performed followed by Tukey’s post-hoc test. Unless otherwise indicated, data are reported as means ± S.D. of at least 3 independent experiments. P < 0.05 was considered significant.

## Results

### PRP modulates TNFα-induced responses in macrophages

**Figure 1:**
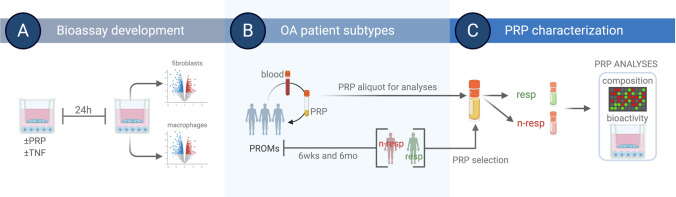
*Study workflow.* (**A**) We evaluated the anti-inflammatory activity of PRP using cocultures of macrophages and fibroblasts treated with PRP without or with TNFα for 24 h. After treatment, RNA was isolated from macrophages and fibroblasts for RNA-seq. (**B**) We collected PRP and patient reported outcomes measures (PROMs, Knee injury and Osteoarthritis Outcome and Numeric Pain Rating Scale) in knee OA patients that received one intra-articular injection of PRP. We defined two patient subtypes based on achievement of minimal clinically important differences at 6 weeks (6 wks) and 6 months (6 mo) after injection, relative to baseline. Based on these criteria, patients that report positive responses (reduced pain) were considered responders (resp), and patients that did not respond to the treatment were considered non-responders (n-resp). (**C**) We compared the composition of PRP selected from resp and n-resp using ELISA and multiplex ELISA. We compared the bioactivity of PRP from resp and n-resp using the coculture system and changes in the expression of specific gene targets. Created with BioRender.com.

We aimed to develop an integrative approach that permits the evaluation of PRP bioactivity in vitro, using PRP from patients with different responses to PRP (see Fig. 1 for study design). First, we established a coculture system of human macrophages and synovial fibroblasts and compared their transcriptional profiles in response to TNFα, alone or in combination with PRP. We selected this coculture system because of the well-known contribution of synovial fibroblasts and macrophages to OA^17,18^, and because this coculture system and the crosstalk of these cells in response to TNFα is also well-established^16^. We performed RNA-seq analyses in 4 independent cocultures (prepared with macrophages and fibroblasts from different tissue donors and using PRP samples from 4 independent donors) treated with 10 ng/ml of TNFα and 10% (vol:vol) of PRP, alone or combined. Macrophages and fibroblasts were co-treated for 24 h and then RNA was isolated from each cell type separately for RNA-seq analyses.

**Figure 2:**
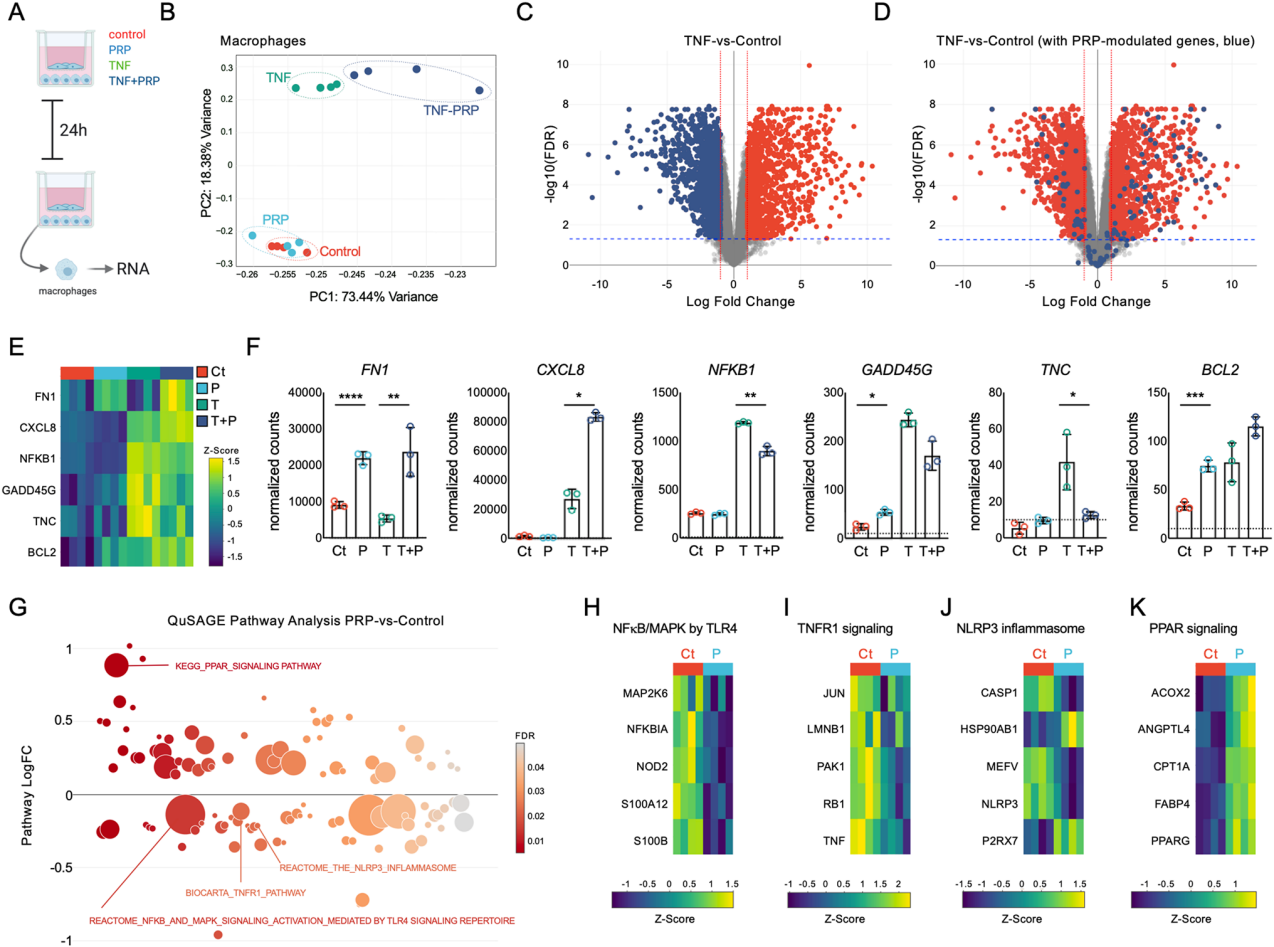
*Transcriptomic analyses of the modulation of TNFα-driven responses by PRP in macrophages.* (**A**) Schematic representation of the experimental workflow. Created with BioRender.com (**B**) Principal component analysis (PCA, using top 1,000 genes) of RNA-seq data from macrophages left untreated, or treated with TNFα and PRP, alone or combined, for 24 h. RNA-seq analysis is based on 4 independent coculture experiments, with macrophages isolated from 4 different tissue donors and PRP prepared from 4 different subjects. (**C**) Volcano plot of differentially expressed genes comparing TNFα-treated macrophages to untreated controls. Colored dots correspond to genes with significant changes greater than 1 log2-fold expression change (FDR < 0.05, LogFC > 1). Red indicates increased expression and blue indicates decreased expression after TNFα treatment. (**D**) Volcano plot representing PRP-induced changes in macrophages co-treated with TNFα + PRP. Red dots represent differentially expressed genes (DEGs) induced by TNFα relative to untreated controls, and blue dots represent PRP-induced changes in PRP + TNFα treated cells relative to TNFα-treated macrophages (FDR < 0.05, LogFC > 1). (**E**) Heatmap representation of selected DEGs by RNA-seq, and (**F**) NanoString analyses showing the normalized counts of these same selected genes, confirming changes in the control (Ct), PRP- (P), TNFα- (T) and PRP + TNFα-treated (P + T) macrophages. Dotted lines indicate background signal. *p < 0.05, **p < 0.01, ***p < 0.001, ****p < 0.0001 by *t*-test, calculated using ratio data and the nSolver analysis software. NanoString data is represented as the mean ± S.D (error bars) normalized counts of 3 independent coculture experiments, using macrophages isolated from 3 different tissue donors and PRP prepared from 3 different subjects. Graphs were created with GraphPad Prism 8. (**G**) Representation of the QuSAGE pathway analyses in PRP-treated macrophages relative to untreated controls, showing functional pathways differentially expressed in PRP-treated cells (FDR < 0.05). Heatmap representation of representative genes associated with the (**H**) NF-κB/MAPK, (**I**) TNFR1, (**J**) NLRP3 inflammasome, and (**K**) PPAR signaling pathways. Data was z-score normalized.

Analyses of RNA isolated from macrophages (Fig. 2A) after PRP, TNFα, and TNFα + PRP treatment uncovered unique transcriptional profiles relative to non-treated controls, as shown by the principal component analyses (PCA, Fig. 2B). Relative to non-treated controls, macrophages treated with PRP showed decreased expression of several canonical NF-κB targets and stress-response genes, including *S100A12* and *TNFα*, and pronounced upregulation of *MAP2K6* mRNA. As expected, TNFα-treated cells (Fig. 2C) showed increased expression of canonical NF-κB targets (including *CCL5* and *NFKB1*) and the previously reported TNFα-induced IFN responses^16^, with increased *CXCL10*, *NKG7* and *IL1B* mRNA. Comparisons between PRP + TNFα vs. TNFα-treated macrophages uncovered 559 differentially expressed genes (DEGs, Fig. 2D), including decreased expression of known modulators of inflammation (*JUN*, *IRF4* and *SMAD3*; Supplementary Table S3 for the complete list). NanoString multiplex gene expression analyses confirmed changes in expression of genes identified by RNA-seq analyses in macrophages (Fig. 2E, F, and Supplementary Table S4).

Next, we performed QuSAGE pathway analyses^29^ using RNA-seq data. Comparison of PRP-treated macrophages relative to non-treated controls (Fig. 2G) uncovered changes in NF-κB/MAPK (primarily driven by the reduced expression of *S100A12* and *MAP2K6*, Fig. 2H), decreased expression of the TNFR1 (Fig. 2I) and NLRP3 inflammasome (Fig. 2J) pathways, and increased expression of the PPAR signaling pathway (Fig. 2K, and Supplementary Table S5). TNFα-treated macrophages showed decreased WNT signaling and the expected increased expression of genes in the TNFα pathway relative to non-treated controls (Supplementary Table S6). The comparison between the TNFα + PRP and TNFα groups uncovered seven differentially expressed pathways, including decreased expression of the cholesterol biosynthesis pathway and increased expression of the pyruvate dehydrogenase PDH complex signaling in macrophages (Supplementary Table S7).

### PRP modulates TNFα-induced responses in synovial fibroblasts

**Figure 3:**
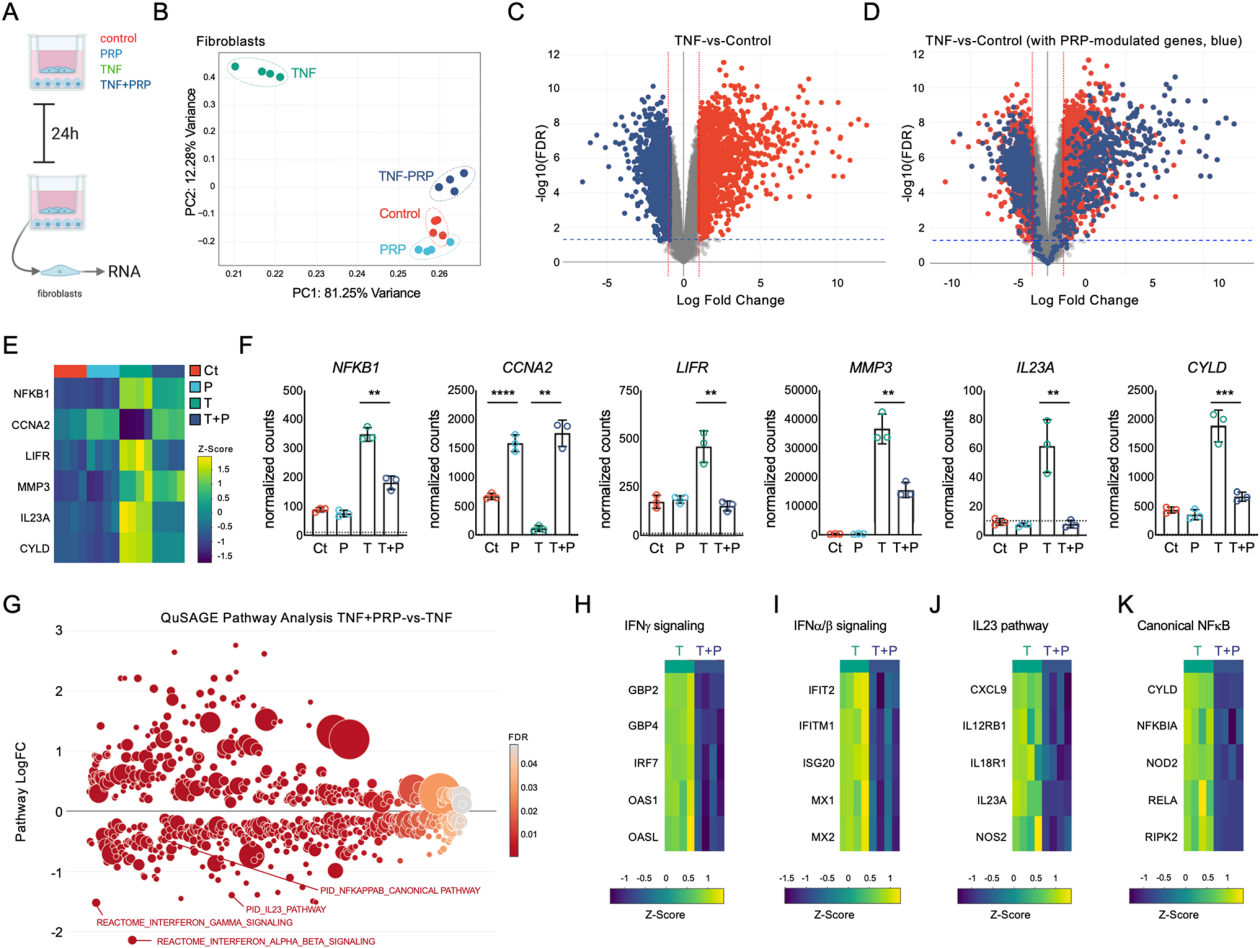
*Transcriptomic analyses of the modulation of TNFα-driven responses by PRP in fibroblasts.* (**A**) Schematic representation of the experimental workflow. Created with BioRender.com (**B**) Principal component analysis (PCA, using top 1,000 genes) of RNA-seq data from fibroblasts left untreated, or treated with TNFα and PRP, alone or combined, for 24 h. RNA-seq analysis is based on 4 independent coculture experiments, with fibroblasts isolated from 4 different tissue donors and PRP prepared from 4 different subjects. (**C**) Volcano plot of differentially expressed genes comparing TNFα-treated fibroblasts to untreated controls. Colored dots correspond to genes with significant changes greater than 1 log2-fold expression change (FDR < 0.05, LogFC > 1). Red indicates increased expression and blue indicates decreased expression after TNFα treatment. (**D**) Volcano plot representing PRP-induced changes in fibroblasts co-treated with TNFα + PRP. Red dots represent differentially expressed genes (DEGs) induced by TNFα relative to untreated controls, and blue dots represent PRP-induced changes in PRP + TNFα treated cells relative to TNF-treated cells (FDR < 0.05, LogFC > 1). (**E**) Heatmap representation of selected DEGs by RNA-seq, and (**F**) NanoString analyses showing the normalized counts of these same selected genes, confirming changes in the control (Ct), PRP- (P), TNFα- (T) and PRP + TNFα-treated (P + T) fibroblasts. Dotted lines indicate background signal. **p < 0.01, ***p < 0.001, ****p < 0.0001 by *t*-test, calculated using ratio data and the nSolver analysis software. NanoString data is represented as the mean ± S.D (error bars) normalized counts of 3 independent coculture experiments, using fibroblasts isolated from 3 different tissue donors and PRP prepared from 3 different subjects. Graphs were created with GraphPad Prism 8. (**G**) Representation of the QuSAGE pathway analyses in TNFα + PRP-treated fibroblasts relative to TNFα-treated cells, showing functional pathways differentially expressed in TNFα + PRP-treated cells (FDR < 0.05). Heatmap representation of representative genes associated with the (**H**) interferon gamma, (**I**) interferon alpha beta, (**J**) IL23, and (**K**) canonical NF-κB signaling pathways. Data was z-score normalized.

Analyses of RNA isolated from cocultured fibroblasts (Fig. 3A) after PRP, TNFα, and TNFα + PRP treatment also uncovered unique transcriptional profiles, with robust and pronounced differences in TNFα-treated fibroblasts vs. non-treated controls, and remarkable differences between TNFα + PRP and TNFα-treated cells (Fig. 3B). Comparison of fibroblasts treated with PRP relative to non-treated controls uncovered 776 differentially expressed genes, including *IGF1*, *VEGFA*, *CDK1*, *IL1A*, and other genes involved in replication, proliferation, DNA repair, and cell survival. Comparison of TNFα-treated fibroblasts with control cells (Fig. 3C) showed a robust induction of classic inflammatory and NF-κB-responsive genes (including *CCL5*, *NFKBIA*, or *RELA*) as well as up-regulation of IFN signature genes (including *MX1*, *MX2*, *IRF1*, *IRF7* or *SOCS*). Fibroblasts co-treated with PRP and TNFα showed very pronounced differences relative to the TNFα-treated cells, with 2,738 DEGs in PRP + TNFα-treated fibroblasts relative to the TNFα-treated group (Fig. 3D). In agreement with its proposed anti-inflammatory actions, PRP co-treatment modulated and, in many cases, abolished TNFα-driven induction of NF-κB-responsive genes (including *CCL5* or *RELA*) and IFN signatures (with decreased expression of *IRF1*, *MX1* or *MX2*, Supplementary Table S8). NanoString analyses confirmed changes in expression of genes identified by RNA-seq analyses in fibroblasts. See Fig. 3E, F for selected genes and Supplementary Table S9 for a summary of the NanoString normalized counts in fibroblasts.

QuSAGE pathway analyses in PRP-treated fibroblasts relative to untreated controls confirmed the differential modulation of functional pathways relevant to cell cycle, DNA synthesis and cell survival, and also showed repression of ATF4, ATF6, and PI3K/AKT signaling relative to non-treated controls (Supplementary Table S10). Pathway analyses in TNFα-treated cells relative to non-treated controls confirmed the potent upregulation of IFN and NF-κB signatures, as well increased expression of the extrinsic pathway for apoptosis and repression of DNA repair (Supplementary Table S11). Comparison of TNFα + PRP- versus TNFα-treated fibroblasts showed pronounced changes in different functional pathways (Fig. 3G and Supplementary Table S12), including decreased expression of the interferon gamma (Fig. 3H), interferon alpha beta (Fig. 3I), IL23 (Fig. 3J), and NF-κB (Fig. 3K) signaling pathways.

These analyses in macrophages and fibroblasts demonstrated a profound anti-inflammatory effect of PRP, both at baseline and in response to TNFα-induced inflammation. In additional experiments, we treated macrophages and fibroblasts with IL-1β alone or combined with PRP for 24 h. As shown is Supplementary Figure S1, PRP co-treatment also down-modulated responses to IL-1β in our coculture system, indicating that the immunomodulatory and anti-inflammatory actions of PRP are not cytokine-specific. Experiments using macrophage monocultures treated with TNFα and PRP suggested that the PRP actions on macrophages did not depend upon the fibroblast-macrophage crosstalk (Supplementary Figure S2).

### The PRP modulation of TNFα-driven responses is concentration- and time-dependent

**Figure 4:**
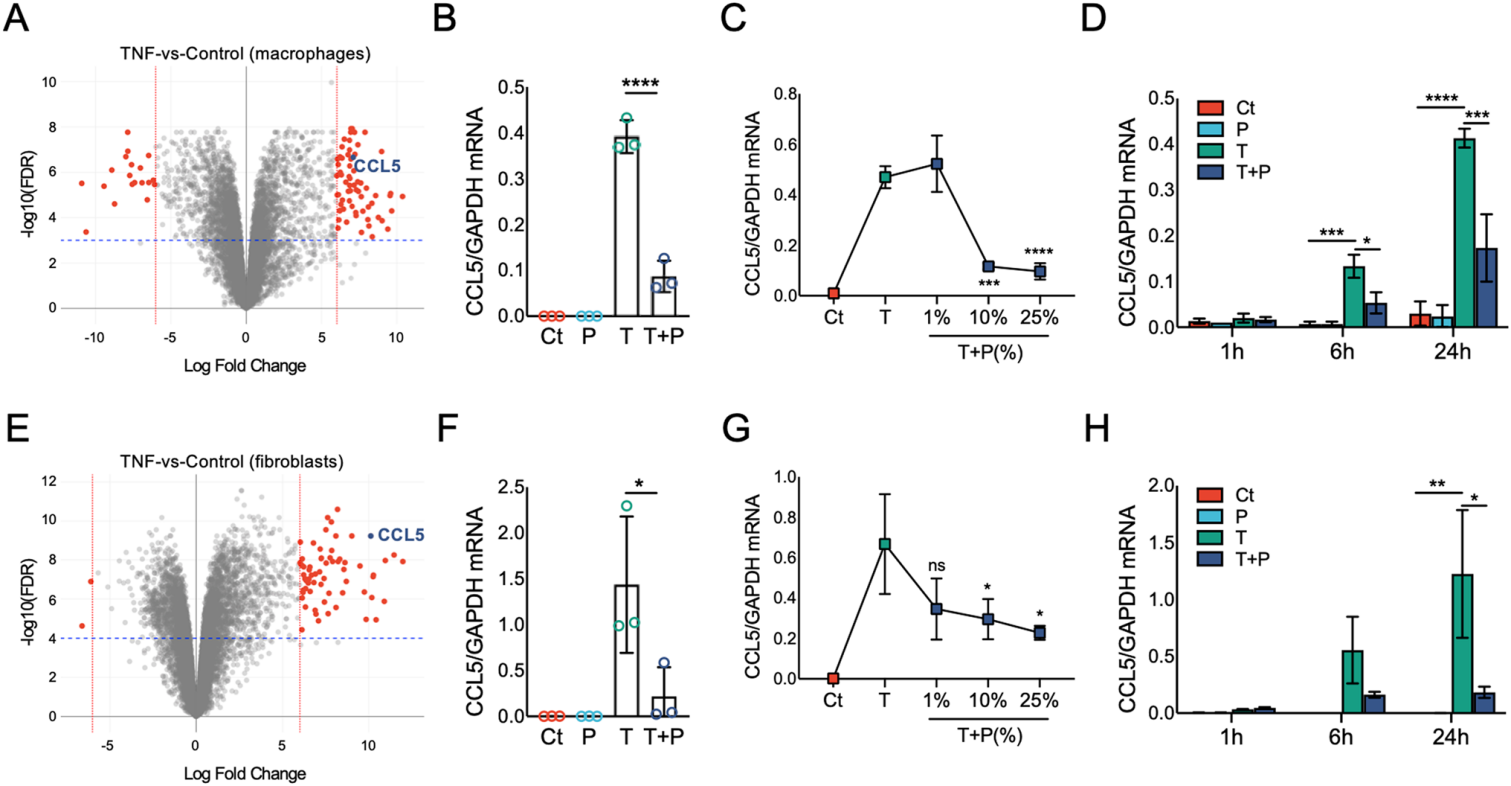
*Dose- and time-dependent PRP modulation of the TNFα-induced CCL5 expression in macrophages and fibroblasts.* (**A**) Volcano plot of differentially expressed genes comparing TNFα-treated macrophages to untreated controls. Genes with significant changes (FDR < 0.0001) and logFC > 6 are in red. *CCL5* is highlighted in blue. (**B**) RTqPCR analyses in macrophages left untreated (Ct) or treated with TNFα (T) and 10% v:v PRP (P) alone or combined (T + P) for 24 h. ****p < 0.001 by ANOVA followed by Tukey’s post-hoc test. (**C**) RTqPCR analyses in macrophages left untreated (Ct) or treated with TNFα alone (T) or combined (T + P) with different concentrations (%, vol:vol) of PRP for 24 h. ***p < 0.001, ****p < 0.0001 versus TNFα by ANOVA followed by Tukey’s post-hoc test. (**D**) RTqPCR analyses in macrophages left untreated (Ct), or treated with TNFα (T), 10% (v:v) PRP (P), or TNFα + PRP (T + P) for 1 h, 6 h and 24 h. *p < 0.05, ***p < 0.001, ****p < 0.0001 by ANOVA followed by Tukey’s post-hoc test. **(E**) Volcano plot of differentially expressed genes comparing TNFα-treated fibroblasts to untreated controls. Genes with significant changes (FDR < 0.0001) and logFC > 6 are in red. *CCL5* is highlighted in blue. (**F**) RTqPCR analyses in fibroblasts left untreated (Ct) or treated with TNFα (T) and 10% v:v PRP (P) alone or combined (T + P) for 24 h. *p < 0.05 by ANOVA followed by Tukey’s post-hoc test. (**G**) RTqPCR analyses in fibroblasts left untreated (Ct) or treated with TNFα alone (T) or combined (T + P) with different concentrations (%, vol:vol) of PRP for 24 h. ns = not significant, *p < 0.05 versus TNFα by ANOVA followed by Tukey’s post-hoc test. (**H**) RTqPCR analyses in fibroblasts left untreated (Ct), or treated with TNFα (T), 10% (v:v) PRP (P), or TNFα + PRP (T + P) for 1 h, 6 h and 24 h. *p < 0.05, **p < 0.01, by ANOVA followed by Tukey’s post-hoc test. RTqPCR data is represented as mean ± S.D (error bars) of 3 independent coculture experiments, using macrophages and fibroblasts isolated from 3 different tissue donors and PRP prepared from 3 different subjects. RNA-seq is based on 4 independent coculture experiments, with macrophages and fibroblasts isolated from 4 different tissue donors and PRP prepared from 4 different subjects. RTqPCR graphs created with GraphPad Prism 8.

We next aimed to identify specific gene targets that can be used as readouts for the cellular responses to PRP in macrophages and fibroblasts. Our transcriptomic analyses showed that PRP strongly modulated the expression of canonical NF-κB targets in macrophages and fibroblasts, in agreement with studies that reported that the modulation of NF-κB by PRP is cell- and PRP composition-dependent^34–36^. We identified *CCL5* (*RANTES*) as one of the direct canonical NF-κB targets^37,38^ with the most robust (logFC > 6 and FDR < 0.0001) increased expression upon TNFα treatment in both macrophages and fibroblasts (Fig. 4A, E). *CCL5* also displayed PRP-dependent inhibition in these cells, particularly evident in TNFα-treated fibroblasts (Supplementary Tables S3 and S8). RTqPCR analyses in cocultures confirmed the TNFα-induced expression of *CCL5* in macrophages, with reduced expression in TNFα + PRP-treated cells (Fig. 4B). Cocultures of macrophages and fibroblasts with TNFα, alone or combined with different concentrations (% vol:vol) of PRP for 24 h showed that *CCL5* displayed a robust dose–response to PRP in macrophages (Fig. 4C). RTqPCR analyses of time-course experiments in cocultures treated with TNFα, alone or combined with 10% PRP for 1, 6 and 24 h showed time-dependent induction of *CCL5* mRNA upon TNFα treatment in macrophages, and inhibition by PRP co-treatment at 6 and 24 h after treatment (Fig. 4D). Similarly, qPCR analyses in cocultures confirmed the TNFα-dependent induction of *CCL5* and reduced expression after co-treatment with PRP in fibroblasts (Fig. 4F). In agreement with the results in the cocultured macrophages, the responses to PRP were also concentration- (Fig. 4G) and time-dependent (Fig. 4H) in fibroblasts. Other tested genes in macrophages and fibroblasts displayed less robust dose- or time-dependent responses or were only reliable in one of the cell types (Supplementary Figure S3).

Based on these results, we selected *CCL5* as a target, and 10% PRP and 24 h stimulation in our coculture conditions for follow-up analyses and evaluation of PRP bioactivity in patient samples.

### Differences in bioactivity in PRP from OA patients with poor clinical outcomes

**Figure 5:**
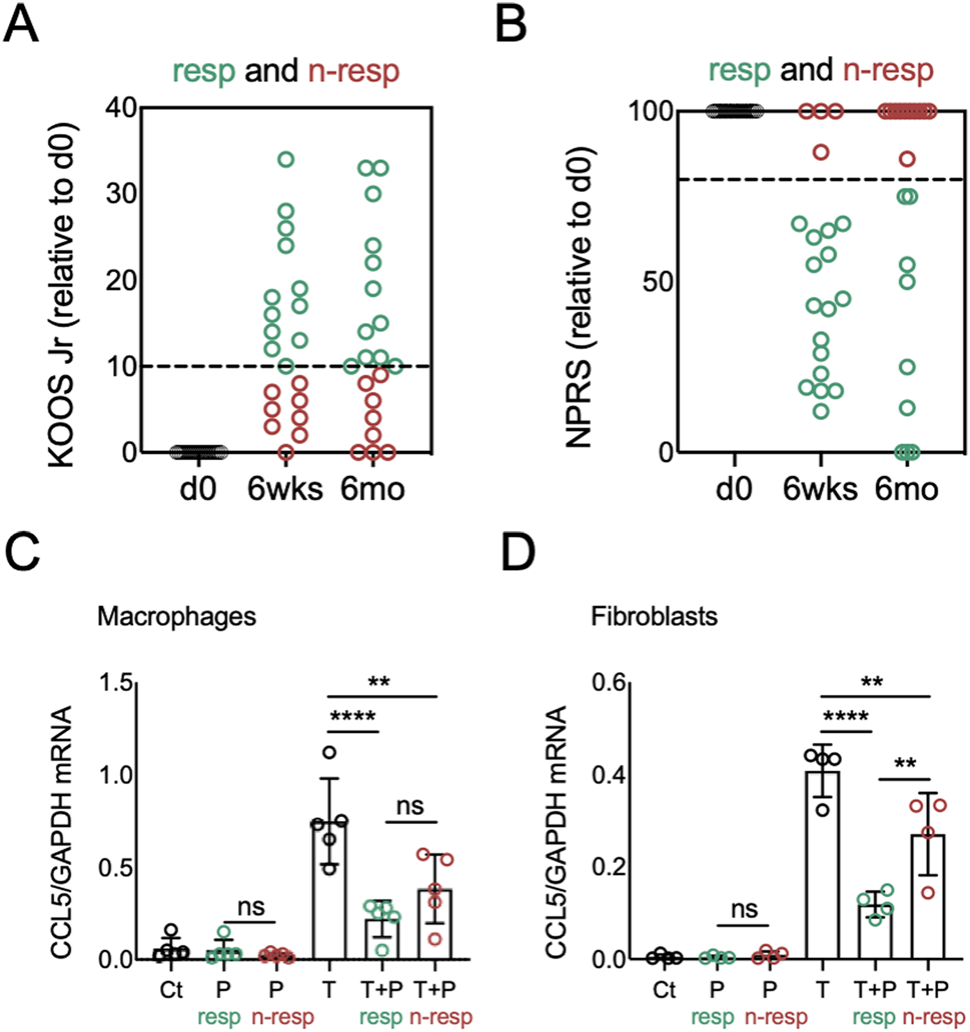
*Evaluation of the bioactivity of PRP from patients classified as responders and non-responders using patient-reported outcomes*. Responders (resp, green) and non-responders (n-resp, red) to PRP treatment identified using (**A**) Knee injury and Osteoarthritis Outcome (KOOS JR) Score and (**B**) Numeric Pain Rating Scale (NPRS) Score in patients with completed questionnaires at baseline (d0) and the 6 weeks (6 wks) and 6 months (6 mo) follow up time-points. For selection of PRP for analyses, patients were categorized as responders or non-responders based on achievement of minimal clinically important difference (MCID) thresholds (dotted lines) of 10-point improvement in KOOS JR and 20% improvement in NPRS at 6 months after PRP injection. Scores are represented as changes relative to self-reported baseline levels (d0) for each patient and each score. RTqPCR analyses of *CCL5* mRNA in (**C**) macrophages and (**D**) fibroblasts left untreated (Ct) or treated with TNFα (T), PRP (P) or TNFα + PRP (T + P) for 24 h, using PRP from responders or non-responders. RTqPCR data is represented as mean ± S.D (error bars) of 5 (macrophages) and 4 (fibroblasts) independent experiments. **p < 0.01, ****p < 0.0001 by ANOVA followed by Tukey’s post-hoc test. ns = not significant. Graphs were created with GraphPad Prism 8.

Next, we aimed to assess if the in vitro bioactivity from the PRP obtained from patients reporting improved pain outcomes after PRP treatment is different than the bioactivity of PRP from patients reporting poor outcomes. To do this, we enrolled 51 patients that received one single leukocyte-poor PRP injection (3.7 ± 0.7 ml, prepared using the Arthrex ACP® kit) to treat knee OA. See Table 1 for patient demographics and PRP characteristics. We collected relevant clinical information at baseline, and self-reported Knee injury and Osteoarthritis Outcome (KOOS-JR) and Numeric Pain Rating Scale (NPRS) scores at baseline and at 6 weeks and 6 months after PRP injection. We used these patient-reported outcomes to identify responders (resp) or non-responders (n-resp) to PRP based on achievement of Minimal Clinically Important Difference (MCID) values at 6 months after PRP injection (see Patients and Methods and^21,22^). We selected 6 months as a time-point to define resp and n-resp to PRP and conduct analyses based on studies that evaluated pain scores in knee OA patients in response to PRP treatment at 6 and 12 months^23^. Figure 5A and B show the distribution of outcome metrics over time, representing only patients that completed both questionnaires at baseline (d0, immediately before PRP administration) and at 6 weeks and 6 months after PRP treatment (N = 20).

Using exclusively outcomes collected at 6 months^23^ after treatment relative to baseline, we identified 17 responders and 15 non-responders (Supplementary Table S13). We did not observe significant differences in Whole-Organ Magnetic Resonance Imaging Score (WORMS)^32^, age, sex, BMI, or concentration of platelets in the injected PRP comparing responders and non-responders (Supplementary Table S13). Multiplex ELISA analyses of PRP from responders and non-responders uncovered minor differences in IL-7 protein, but the relative abundance of other analyzed factors that are believed to drive PRP bioactivity^4,39^ was largely similar between responders and non-responders (Supplementary Table S14), including IL1β (*p* = 0.264), TNFα (*p* = 0.602), or VEGF (*p* = 0.770). Similarly, we did not detect significant differences in TGFβ1 protein by ELISA, comparing PRP from responders and non-responders (Supplementary Figure S4).

Next, we used our in vitro bioassay to evaluate differences in the bioactivity of PRP from responders and non-responders. We selected PRP samples from 5 patients that met the “responder” MCID criteria at 6 months^23^ for both NPRS and KOOS-JR scores, and we compared them with PRP samples from 5 patients that were categorized as non-responders at 6 months for both scores. We prepared cocultures of macrophages and fibroblasts using cells from 5 independent blood and tissue donors, respectively. We treated the cocultures with TNFα, alone or combined with 10% (v:v) PRP from responders or non-responders. At 24 h after treatment, we analyzed the *CCL5* mRNA expression by RTqPCR in macrophages and fibroblasts. The RNA isolated from the cocultured fibroblasts from one experiment was lost during processing. While the co-treatment with PRP from responders and non-responders was able to inhibit the TNFα-induced *CCL5* expression in macrophages (Fig. 5C) and fibroblasts (Fig. 5D), we detected cell-specific differences. In macrophages (Fig. 5C), we did not detect differences in the PRP-driven inhibition of TNFα-induced *CCL5* expression between responders and non-responders (*p* = 0.4046). In contrast, in fibroblasts (Fig. 5D) the PRP from responders had a more potent inhibitory effect on the TNFα-induced *CCL5* expression (*p* = 0.0017 vs. non-responders). We also evaluated the effects of PRP from responders and non-responders in the TNFα-induced *PPBP* and *IL23A* mRNA expression in macrophages and fibroblasts, respectively (Supplementary Figure S5). In macrophages (Supplementary Figure S5A), the PRP from responders synergized with TNFα to significantly increase *PPBP* expression (*p* = 0.0146 vs. TNFα, and *p* = 0.0071 vs. control), whereas the PRP from non-responders did not (*p* = 0.7785 vs. TNFα, and *p* = 0.6000 vs. control). In fibroblasts, co-treatment with PRP from responders and non-responders inhibited the TNFα-induced *IL23A* expression, and we did not detect differences between cells co-treated with TNFα and PRP from responders and cells co-treated with TNFα and PRP from non-responders (*p* = 0.5935, Supplementary Figure S5B).

Taken together, our results provide comprehensive molecular-level data on the anti-inflammatory actions of PRP in macrophages and synovial fibroblasts in vitro, and show that the integration of clinical and multi-omics data can help us identify differences in PRP bioactivity that may be associated with clinical outcomes. Our bioassay also highlighted the complex cell- and gene-specific actions of PRP that need to be taken into account for the development of targeted therapeutic approaches.

## Discussion

Here we show that integration of clinical data and molecular analyses in a relevant in vitro model that mimics joint inflammation can be effectively used to identify differences in PRP bioactivity and may provide information about its association with clinical outcomes. Using this approach, we characterized the anti-inflammatory actions of PRP in fibroblasts and macrophages treated with TNFα, and we identified differences in bioactivity in PRP samples selected from knee OA patients with different clinical responses to the PRP treatment.

Positive outcomes after PRP treatment for OA are believed to be primarily related to the anti-inflammatory effects of PRP^4,14^. OA patients receiving leukocyte-poor PRP reported superior Western Ontario and McMaster Universities Osteoarthritis Index (WOMAC) scores compared to patients treated with hyaluronic acid^40^ or placebo^41^, in agreement with previous reports comparing PRP- and placebo-treated knee OA patients^42^. Similarly, leukocyte-poor PRP was more effective than conventional therapy (acetaminophen) to treat patients with early knee OA^43^. However, the functional impact of PRP to treat OA is still not conclusive because of the variable clinical results, which are believed to be related to both the variable PRP composition and to patient-intrinsic factors^3,15^.

In this study we did not aim to address PRP efficacy, and we relied on patient-reported outcomes exclusively to stratify patients into responders and non-responders and select PRP samples for downstream analyses. We did observe variable responses to PRP that are consistent with the literature, with patients clearly meeting MCID improvement thresholds and patients that did not show a clinically significant improvement following treatment. In our patient cohort, the variation in clinical responses was not associated with disease severity, in agreement with previous reports^44^. Similarly, we did not detect differences in sex, age, BMI, or MRI scores between patients that we defined as responders and non-responders. It is worth noting that we exclusively analyzed MRI images obtained before PRP administration, and we did not evaluate changes in cartilage morphology in response to PRP treatment with post-treatment MRI. It is conceivable that a longitudinal follow up of patients after PRP treatment could uncover patient-specific changes associated with responses to PRP. Therefore, there is still a need for larger clinical studies that comprehensively evaluate patient-specific features and their association with responses to PRP. These studies should include MRI and other forms of longitudinal evaluation that permit a comprehensive assessment of responses to the treatment over time, to identify good candidates for these emerging therapies.

Technique-^45^ and patient-dependent^46,47^ factors are known to contribute to variable PRP composition and are believed to significantly contribute to the variability in clinical outcomes, as also indicated by the differences in efficacy between leukocyte-rich and -poor PRP^40^. In this study we aimed to minimize technical variability using a single commercially available kit that results in autologous conditioned plasma (ACP, a form of leukocyte-poor PRP with relatively low enrichment in platelets), and standardizing the treatment protocol (single injection, equivalent volume injected). However, we still detected significant variability in the responses to PRP, which were not associated with age, sex, number of platelets in the PRP, or other demographics or technical metrics. Comparing the PRP from responders and non-responders, we did not detect differences in IL-1RA, VEGF, EGF, TGFβ or other cytokines that are classically associated with PRP activity^4,39^. We did find a minor increase in IL-7 in the PRP from responders relative to non-responders. IL-7 has well-described pleiotropic roles in different cell subsets^48–50^ and has been implicated in pain responses^51,52^. However, a functional connection between IL-7 and the PRP bioactivity in vitro, or with clinical outcomes in OA patients, was not established. Further, in our study we did not detect significant differences in the presence of platelets or leukocyte subtypes in the whole blood and resulting PRP of responders and non-responders that could explain these differences in IL-7. Follow up studies should evaluate the relative contribution of these molecules to the PRP activity in a cell- and tissue-specific context. These studies should focus on the combinatorial ratios and potential synergistic interactions of different bioactive molecules, including factors classically associated with PRP activity, instead of relative changes in the levels of one single factor. Such studies are required to better understand how the variable composition of PRP impacts clinical outcomes in OA patients and other orthopedic conditions.

Studies in preclinical models and in vitro systems indicated that platelet products can lead to some structural improvement, promote chondrogenesis and facilitate tissue repair^53,54^. These studies highlighted the immunomodulatory and anti-inflammatory actions of platelet-derived factors in different cell types^35,55,56^, and studies in knee OA patients have shown that the clinical benefits of PRP are likely related to pain-modifying anti-inflammatory actions^14,23^. We tested the bioactivity of PRP using a well-established coculture of macrophages and fibroblasts^16^. These cells contribute to the inflammatory responses in OA^17,18^ and our coculture reproduces the cellular crosstalk that occurs in inflamed synovial tissues^16^. PRP treatment or co-treatment with TNFα modulated transcriptional networks that include *SMAD3*, *EGR1*, or *IRF7* in macrophages and fibroblasts, with a remarkable effect on IFN signatures and canonical NF-κB targets, consistent with other studies^35,55,56^. Specifically, in fibroblasts, co-treatment with PRP lead to a pronounced inhibition of the TNFα-induced IFNα/β and IFNγ signaling, with well-described roles in tissue homeostasis and inflammatory pathologies (for review, see^57^). PRP treatment also led to the inhibition of NF-κB-related pathways in macrophages and fibroblasts, including TRAF6-mediated NF-κB activation, TAK1 activation of NF-κB, or NLRP3 inflammasome signaling pathways. NF-κB is known to contribute to OA disease^58,59^ and to pain responses in different inflammatory contexts^60^. Thus, these results are consistent with the proposed anti-inflammatory and immunomodulatory actions of PRP. However, the functional impact of the PRP-driven anti-inflammatory actions requires further investigation, particularly because a fine-tuned modulation of these signaling pathways is required for homeostasis, and because the effects of therapeutic blockade in pathological conditions are likely cell/tissue-specific^57,59^. Taken together, our results further highlighted the complex cell- and gene-dependent actions of PRP in joint-derived cells, reinforcing the notion that studies that address this complexity are required to enable the efficacious targeted application of PRP to knee OA and other musculoskeletal conditions.

Our study has limitations. The targeted evaluation of PRP in vitro uncovered some differences in PRP bioactivity comparing responders and non-responders, but also highlighted the need for using larger group sizes and a wide array of gene targets to capture subtle changes in bioactivity, and to further understand gene- and cell-dependent responses to PRP. The small patient sample size, the lack of a placebo/control group, and the relatively short follow-up time-points used to identify responders and non-responders did not allow us to truly assess the efficacy of PRP as a treatment for OA. The limited set of outcome measures used in this study is also a limitation. Future studies should include placebo controls and longer follow-up time-points, as well as additional metrics for pain, physical function, quality of life, global assessment of the target joint, and adverse effects. Further, using MRI exclusively at baseline may not provide enough information about patient/joint-specific factors associated with clinical responses to PRP, particularly considering that PRP treatment can modulate symptoms independent of the initial disease severity^44^. Studies that incorporate longitudinal imaging assessment of patients receiving PRP injections could provide additional information about the variable responses to PRP treatment for knee OA. Additionally, while we believe that we have used a relevant and sensitive bioassay for the in vitro evaluation of PRP activity, we are aware that a coculture system with macrophages and synovial fibroblasts does not fully capture the complexity of the joint milieu, that short-term stimulation with cytokines does not capture the more chronic, long-term impact of inflammation in OA, and that in addition to transcriptomics, proteomics analyses should be used to evaluate the functional impact of PRP treatment.

Our results provide novel and relevant insight into the actions of PRP in modulating joint inflammation at a transcriptomics level and establish an integrative workflow that may help us comprehensively evaluate differences in PRP bioactivity associated with clinical outcomes, and therefore identify good candidates for PRP treatment. Larger studies that integrate clinical data with multi-omics analyses are needed to further establish the efficacy of PRP for the targeted treatment of knee OA and other orthopedic conditions, and to identify specific characteristics of PRP that are predictive of optimal response to treatment.

## Supplementary Information

Supplementary Information 1.

Supplementary Information 2.

Supplementary Information 3.

Supplementary Information 4.

Supplementary Information 5.

Supplementary Information 6.

Supplementary Information 7.

Supplementary Information 8.

Supplementary Information 9.

Supplementary Information 10.

Supplementary Information 11.

## Data Availability

The data that supports the findings of this study are available from the corresponding author upon reasonable request. The RNA-seq sequencing data have been deposited at the GEO database with accession code GSE157364.
