# Supplementary material for: In vitro responses to platelet-rich-plasma are associated with variable clinical outcomes in patients with knee osteoarthritis
Source: Sci Rep. 2021 Jun 1;11:11493. doi: 10.1038/s41598-021-90174-x (PMC8169703; doi:10.1038/s41598-021-90174-x)
Supplement: Supplementary file 11 — Supplementary Information 11. [file 41598_2021_90174_MOESM11_ESM.pdf]

Supplementary Materials for

In vitro responses to platelet-rich-plasma are associated with variable clinical outcomes in patients with knee osteoarthritis

Habib Zahir[1,2†], Bijan Dehghani[1†], Xiaoning Yuan[1,3], Yurii Chinenov[1,4], Christine Kim[1,5], Alissa Burge[1], Reyna Bandhari[1], Daniel Nemirov[1], Patrick Fava[1], Peter Moley[1,6], Hollis Potter[1], Joseph Nguyen[1], Brian Halpern[1,6], Laura Donlin[1,7], Lionel Ivashkiv[1,4], Scott Rodeo[1,6‡], Miguel Otero[1,6,7‡*]

[†]Habib Zahir and Bijan Dehghani contributed equally to this work

[‡]Scott Rodeo and Miguel Otero contributed equally to this work

Affiliations: [1]Hospital for Special Surgery, New York, NY; [2]New York Institute of Technology, Old Westbury, NY; [3]NewYork-Presbyterian Hospital, New York, NY; [4]The David Z. Rosensweig Genomics Research Center, New York, NY; [5]Columbia University, New York, NY; [6]HSS Center for Regenerative Medicine, New York, NY; [7]Derfner Foundation Precision Medicine Laboratory, New York, NY

***Corresponding author:** Miguel Otero, PhD. HSS Research Institute, Orthopedic Soft Tissue Research Program, Hospital for Special Surgery, 535 E 70th Street, New York, NY 10021, USA. Tel: 212-774-7561. Email: oterom@hss.edu

This file includes:

Figure S1: PRP modulates the IL-1β-induced responses in a coculture of macrophages and fibroblasts.
Figure S2: PRP modulates the TNF-induced responses in macrophages in monoculture.
Figure S3: Time- and dose-dependent responses in macrophages and fibroblasts.
Figure S4: TGFβ1 protein levels in PRP from responders and non-responders.
Figure S5: Comparison of the bioactivity of PRP from responders and non-responders.
Table S1: Inclusion and exclusion criteria.
Table S2: PCR primers.
Table S13: Comparison of responders and non-responders.
Table S14: Luminex ELISA, comparison of responders and non-responders.

Other Supplementary Materials for this manuscript include the following:

Table S3: RNA-seq analyses, differentially expressed genes in macrophages.
Table S4: NanoString analyses, normalized counts in macrophages.
Table S5: QuSAGE pathway analyses in macrophages, PRP-vs-Control.
Table S6: QuSAGE pathway analyses in macrophages, TNF-vs-Control.
Table S7: QuSAGE pathway analyses in macrophages, TNF+PRP-vs-TNF.
Table S8: RNA-seq analyses, differentially expressed genes in fibroblasts.
Table S9: NanoString analyses, normalized counts in fibroblasts.
Table S10: QuSAGE pathway analyses in fibroblasts, PRP-vs-Control.
Table S11: QuSAGE pathway analyses in fibroblasts, TNF-vs-Control.
Table S12: QuSAGE pathway analyses in fibroblasts, TNF+PRP-vs-TNF.

SUPPLEMENTARY FIGURES

Supplementary Figure S1. PRP modulates the IL-1β-induced responses in a coculture of macrophages and fibroblasts. (**A**) RTqPCR analyses of *IL1B* mRNA in macrophages left untreated (Ct) or treated with 10% PRP (P), 10ng/ml of IL1β (I) or a combination of IL1β +PRP (I+P) for 24h. (**B**) RTqPCR analyses of *IL1B* mRNA in macrophages left untreated (Ct) or treated with 10% PRP (P), 20ng/ml of TNFα (T) or a combination of TNFα+PRP (T+P) for 24h. (**C**) RTqPCR analyses of *MMP3* mRNA in fibroblasts left untreated (Ct) or treated with 10% PRP (P), 10ng/ml of IL1β (I) or a combination of IL1β +PRP (I+P) for 24h. (**D**) RTqPCR analyses of *MMP3* mRNA in fibroblasts left untreated (Ct) or treated with 10% PRP (P), 20ng/ml of TNFα (T) or a combination of TNFα+PRP (T+P) for 24h. Data is represented as the mean ± S.D (error bars) of 3 independent co-culture experiments, using macrophages and fibroblasts isolated from 3 different tissue donors and PRP prepared from 3 different subjects. *p<0.05, **p<0.01, ****p<0.0001 by ANOVA followed by Tukey's post-hoc test.

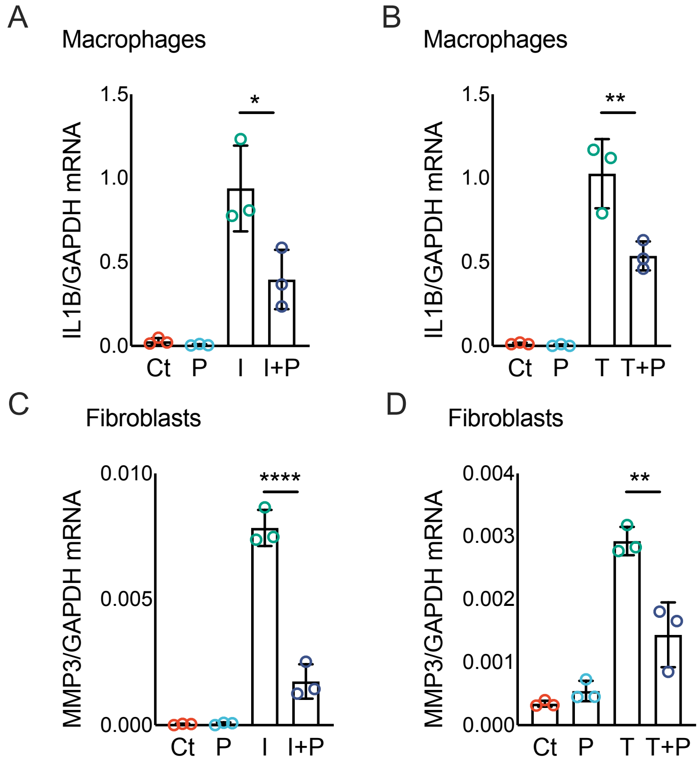

Supplementary Figure S2: PRP modulates the TNFα-induced responses in macrophages in monoculture. RTqPCR analyses of (**A**) *PPBP* and (**B**) *CCL5* mRNA in macrophage monocultures left untreated (Ct) or treated with 10% PRP (P), 20g/ml of TNFα (T) or a combination of TNFα+PRP (T+P) for 24h. Data is represented as the mean ± S.D (error bars) of 2 independent experiments, using macrophages isolated from 2 different tissue donors and PRP prepared from 2 different subjects.

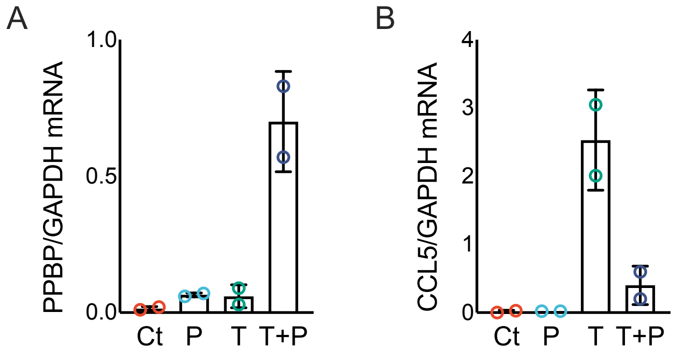

Supplementary Figure S3: Dose- and time-dependent responses in macrophages and fibroblasts. For dose-response experiments, cells were left untreated (Ct) or treated with TNFα (T) alone or combined with 1%, 10% and 25% of PRP (P) for 24 hours. For time-course experiments, cells were left untreated (Ct) or treated with TNFα (T) or 10% v:v PRP (P), alone or combined (T+P) for 1 and 24 hours. RTqPCR analyses of (**A,B**) *CYLD* in fibroblasts, (**C,D**) *CXCL1* in macrophages, (**E,F**) *CXCL3* in macrophages, (**G,H**) *IL23A* in fibroblasts, (**I,J**) *MMP3* in fibroblasts, and (**K,L**) *PPBP* in macrophages. Data are shown as mean ± S.D (error bars) of 3 independent experiments, using macrophages and fibroblasts isolated from 3 different tissue donors and PRP prepared from 3 different subjects. *p<0.05, **p<0.01, ***p<0.001, ****p<0.0001 by ANOVA followed by Tukey's post-hoc test.

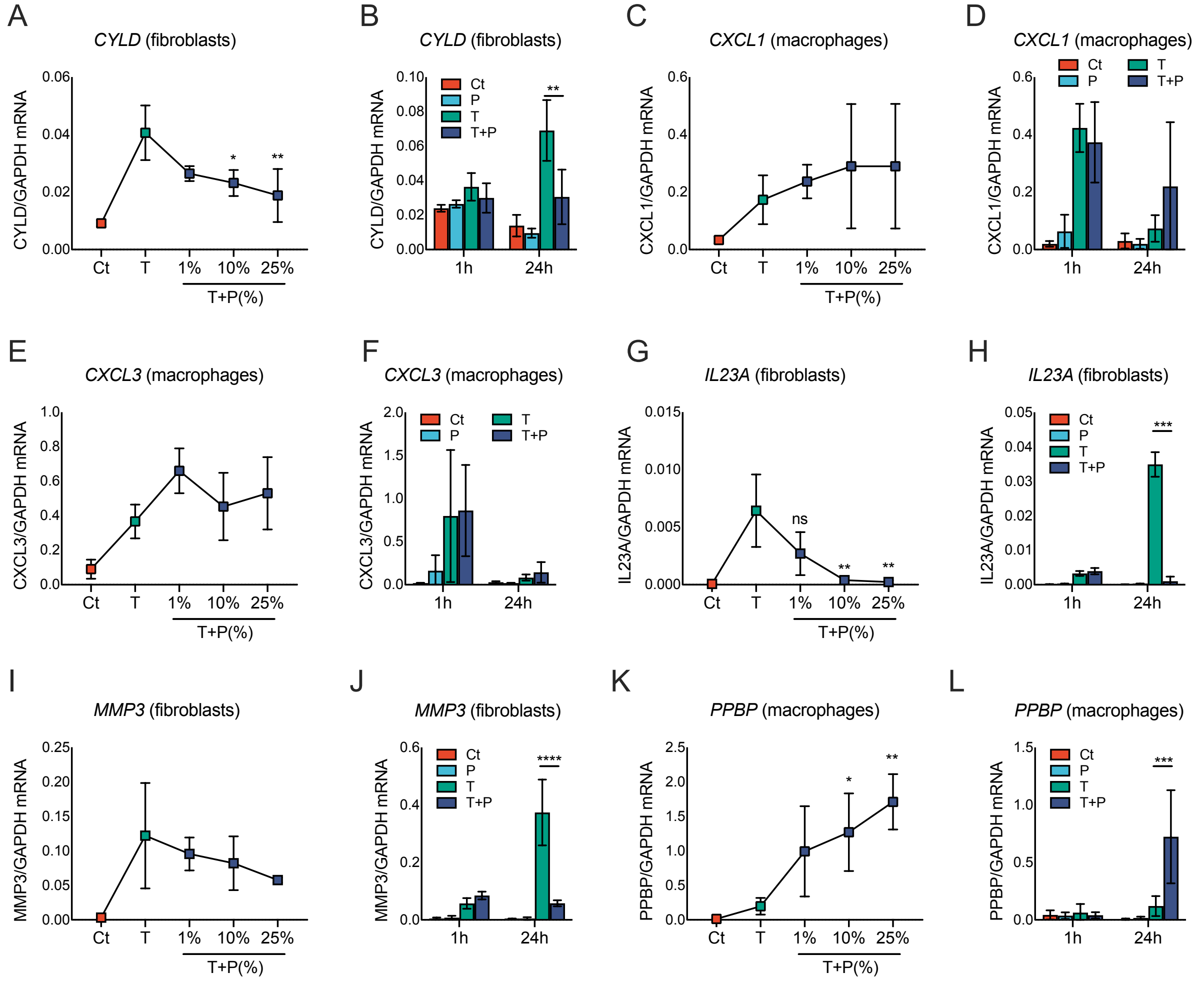

Supplementary Figure S4: TGFβ1 protein levels in PRP from responders and non-responders. The relative abundance of TGFβ1 protein in PRP samples from responders (resp, n=9) and non-responders (n-resp, n=8) was evaluated using ELISA assays. Data was analyzed by Mann-Whitney test.

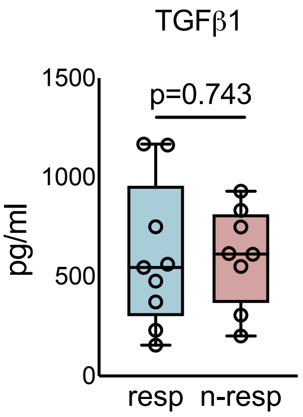

Supplementary Figure S5: Comparison of the bioactivity of PRP from responders and non-responders. RTqPCR analyses of (**A**) *PPBP* mRNA in macrophages and (**B**) *IL23A* mRNA in fibroblasts left untreated (Ct) or treated with TNFα (T), PRP (P) or TNFα+PRP (T+P) for 24 h, using PRP from responders (resp) or non-responders (n-resp). RTqPCR data is represented as mean ± S.D (error bars) of 5 (macrophages) and 4 (fibroblasts) independent experiments. *p<0.05, ****p<0.0001 by ANOVA followed by Tukey's post-hoc test. ns = not significant.

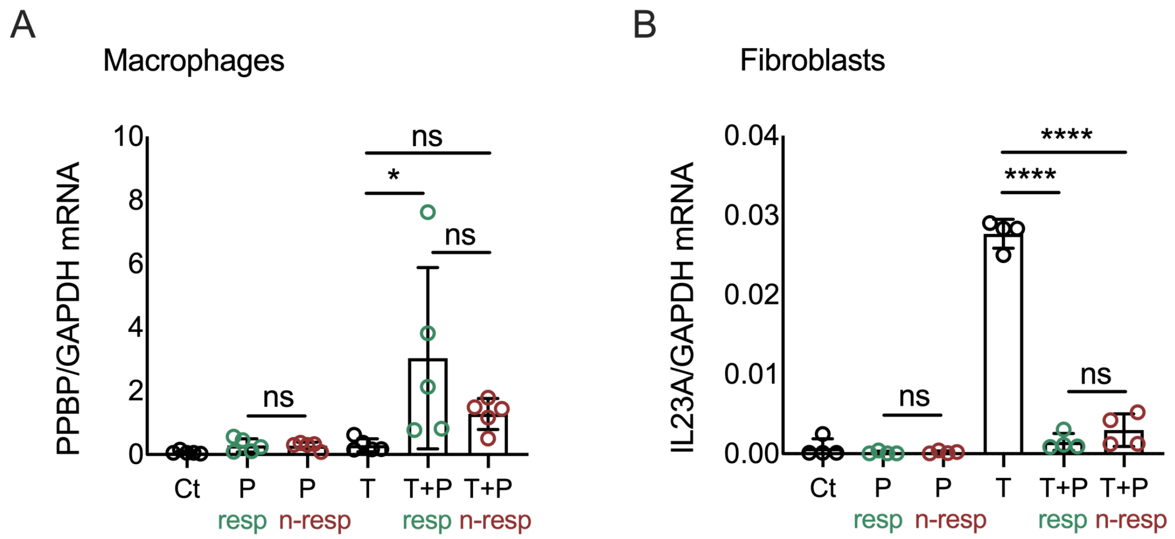

Supplementary Table S1: Inclusion and exclusion criteria (HSS IRB # 2016-0267)

**Inclusion criteria**
Kellgren-Lawrence OA grade 1-3 based on plain radiographs
Age 30-75
Males and females
**Exclusion criteria**
BMI greater than 35
Diagnosis of inflammatory arthritis
Steroid injection within 3 months or hyaluronic acid injection within 6 months
Knee surgery within 1 year
Concurrent use of anti-platelet medications.
Varus malalignment- defined as femoral-tibial angle less than or equal to 0
Valgus malalignment- defined as femoral-tibial angle greater than 10
Evidence of prior complete meniscectomy based on MRI scan
Subchondral insufficiency fracture diagnosed based on bone marrow edema pattern on MRI

Supplementary Table S2: PCR primers and conditions

GENE	NCBI Ref Seq	Forward (5'>3')	Reverse (5'>3')	Anneal
IL23A	NM_016584.3	ACACATGGATCTAAGAGAAGAGGG	AGAGAAGGCTCCCCTGTGAA	60
CCL5	NM_002985.3	GAGGCTTCCCCTCACTATCC	CTCAAGTGATCCACCCACCT	60
CXCL1	NM_001511.4	CCAGCTCTTCCGCTCCTC	CACGGACGCTCCTGCTG	60
CXCL3	NM_002090.2	AGATACTGAACAAGGGGAGCAC	CCTTTCCAGCTGTCCCTAGA	60
IL1B	NM_000576.3	CTCAAGTGATCCACCCACCT	CTCAAGTGATCCACCCACCT	60
MMP3	NM_002422.5	CCCATATATGCCTGCTGTCC	ACAGGTTGATTCCTGGTCA	60
PPBP	NM_002704.3	CTTGGCGAAAGGCAAAGAGG	TCAGTGTGGCTATCACTTCGAC	60
CYLD	NM_015247.2	TTGGTGTGGACATGGATAACCC	CTCCTTTCCTGCGTCACACT	60
GAPDH	NM_002046.7	ATCAAGAAGGTGGTGAAGCA	GTCGCTGTTGAAGTCAGAGGA	60

Supplementary Table S13: Comparison of responders (n=17) vs. non-responders (n=15) to PRP.

	responders	non-responders	p value*
**Sex (male:female)**	7:10	9:6	0.4795 [a]
**Age (years, mean ± s.d.)**	59.53 ± 8.91	57.07 ± 10.97	0.4890
**BMI (kg/m^2, mean ± s.d.)**	25.96 ± 4.11	24.77 ± 3.24	0.3824
**K-L Score (mean ± s.d.)**	1.88 ± 0.72	1.92 ± 0.86	0.8711
**WORMS Score (mean ± s.d.)**	35.00 ± 21.39	38.93 ± 25.76	0.6838
**Platelets (x10^3/ml, mean ± s.d.)**	365.20 ± 71.26	386.00 ± 94.94	0.5124
**Leukocytes (x10^3/ml mean ± s.d.)**	1.01 ± 0.59	1.36 ± 0.67	0.1226

***by *t*-test; [a]by Fisher's exact test**

Supplementary Table S14: Luminex ELISA analyses comparing PRP from responders (resp) vs. non-responders (n-resp).

	N (valid:missing)		Median		Range		Test Statistics	
	**resp**	**n-resp**	**resp**	**n-resp**	**resp**	**n-resp**	**Mann-Whitney U**	**exact P value**
**EGF**	16:1	14:1	96.1	140.3	290.1	284.8	88	0.334
**FGF2**	16:1	15:0	468.6	451.3	477.6	387.7	111	0.74
**IL1α**	16:1	15:0	37.2	23.4	166.8	114.4	94.5	0.318
**IL1β**	16:1	15:0	1.0	1.6	13.8	8.9	91.5	0.264
**IL1RA**	16:1	14:1	1.6	30.3	331.8	478.7	94	0.473
**IL6**	17:0	15:0	0.0	3.2	17.6	23.2	107.5	0.455
**IL7**	15:2	13:2	8.7	3.1	17.8	16.7	50	**0.029**
**IL8**	16:1	14:1	5.2	7.0	12.5	40.1	96.5	0.525
**IL10**	16:1	15:0	0.8	0.0	56.9	508.2	107.5	0.626
**MCP1**	16:1	15:0	160.9	168.5	201.9	166.7	103	0.52
**TNFα**	17:0	15:0	28.3	27.4	33.6	36.8	113.5	0.602
**VEGF**	16:1	15:0	78.9	76.4	268.0	390.8	112.5	0.770
